# Prevalence and Determinants of Opportunistic Infections in HIV Patients: A Cross-Sectional Study in the City of Semarang

**DOI:** 10.4314/ejhs.v32i4.18

**Published:** 2022-07

**Authors:** Sutini, Sri Ratna Rahayu, Muhamad Zakki Saefurrohim, Moch Thoriq Assegaf Al Ayubi, Herlina Wijayanti, Anggun Dessita Wandastuti, Dani Miarso, Mustika Suci Susilastuti

**Affiliations:** 1 Universitas Negeri Semarang, Semarang, Indonesia; 2 Universitas Islam Negeri Syarif Hidayatullah, Jakarta, Indonesia; 3 Semarang Health Office; 4 Universitas Diponegoro, Semarang, Indonesia

**Keywords:** HIV patients, Opportunistic Infections, risk factors, cross-sectional

## Abstract

**Background:**

Opportunistic infection (OI) is the most significant complication of the human immunodeficiency virus (HIV). Differences in the characteristics of HIV patients make the prevalence of Opportunistic infection different between regions. The study aimed to identify variables associated with OI incidence among HIV-infected patients in Semarang City, Indonesia.

**Methods:**

This study uses secondary data sourced from special HIV surveillance for 2019–2021 with a cross-sectional method. 1362 HIV patients with variables health care facilities; year of diagnosis; area of residence; age; sex; pregnancy status; occupation; risk factors; risk group determined based on purposive sampling were included in the chi-square analysis and logistic regression.

**Results:**

This study showed 12.3% (n=167) of HIV patients experienced OI, where OI was more common in HIV patients with risk groups of sex workers (28.70%), high-risk partners (18.60%), and Male Sex with Men (MSM) (15.40). The most common types of OI were tuberculosis infection (43%), candidiasis (21%), and diarrhea (9%). Age was the variable most associated with the incidence of OI (p-value 0.001).

**Conclusions:**

Age groups 45–54 years and 55–64 years have the most influential association with Opportunistic infection incidence in HIV patients, so planning an appropriate intervention program for this subpopulation is necessary.

## Introduction

HIV and AIDS have become a global emergency, one of the most life-threatening infectious diseases, and a severe problem. Without treatment, HIV will gradually destroy the immune system and AIDS (1,2). Globally, 35 million people live with HIV, and 19 million people do not know their HIV-positive status. In Asia, except for Thailand and northern India, most of the HIV infection rate in the general population is less than 1%. In 2012, an estimated 350,000 people in the Asia-Pacific region were newly infected with HIV, of which about 64% were men (3).

Opportunistic Infections (IO) occur due to immune suppression and are believed to be responsible for the development of HIV-related morbidity and mortality (3,4). The most common OI's in HIV-infected patients in Vietnam are oral candidiasis, tuberculosis, wasting syndrome, lower respiratory tract infections, cryptococcosis, and penicilliosis (5). OI are classified according to their severity as WHO clinical stages I to IV. More severe infections are thought to be associated with a poor disease prognosis, and their severity depends on the conditions of exposure to the pathogen, the virulence of the pathogen, and the status of the immune system (6).

The HIV and AIDS epidemic is also a problem in Indonesia, the country with the fifth-highest risk of HIV and AIDS in Asia. Since the first report, new HIV cases have been reported every year. From the first findings in 1987 to December 2018. HIV/AIDS was reported by 460 people (89.5%) in 514 districts and cities in all provinces in Indonesia. As of December 2018, the cumulative number of reported HIV infections was 327,282 (51.1% of the estimated 640,443 in 2016). The cumulative number of people infected with HIV from 1987 to December 2018 was 1.14065 (7,8).

Research conducted in East Java showed that the majority of the AIDS patients in East Java in this sample were male (70.74%), belonged to the adults (26–45 years old) group (62.65%), and worked as employees/laborers (46.08%). In terms of sexual orientation, the majority were heterosexuals (81.81%) or homosexuals (10.63%). The majority of patients experienced one type of opportunistic infection (46.08%), with histoplasmosis (48.77%) and tuberculosis (TB) (42.62%) as the most frequently experienced opportunistic infections (9). Another study in Surabaya showed that heterosexual transmission is a common risk factor in patients. The most prevalent opportunistic infections found in patients were oral candidiasis (58.6%), followed by pulmonary tuberculosis (41.4%) and pneumonia/PCP (41.4%). Other infections found were toxoplasmosis, chronic diarrhea, cytomegalovirus, TB meningitis, hepatitis C, amoebiasis, and cerebritis. Opportunistic infections occurred more often in age ≥40 years and increased as clinical stadiums got worse (10). Based on the description above, this study was conducted to identify variables associated with OI incidence among HIV-infected patients in Semarang, Indonesia.

## Materials and Methods

**Data source and subjects**: This study uses secondary data from the HIV surveillance of the Semarang City Health Office. This is a Semarang city-scale survey with a cross-sectional and non-interventional design. The population includes HIV patients who are recorded on the *Sistem Informasi HIV-AIDS dan IMS* (SIHA) electronic record in 2019 - 2020. The data collection was carried out by using interviews, measurements, and examinations.

**Dependent variable**: opportunistic infections declared by health workers

**Independent variables**: Health care facilities were health facilities that report HIV cases categorized by a hospital, public health center (puskesmas), community health center (balkesmas), and Civil Society Organizations (CSO). Year of diagnosis was the year when the respondent was diagnosed with HIV, which was categorized by the years 2019, 2020, and 2021. The region was the residence of respondents who were grouped based on the Semarang area and Outside Semarang. Age was categorized as <15 years, 15–34 years, 35–44 years, 45–54 years, 55–64 years, and >64 years, pregnant status for women who are categorized as yes and no, and employment was grouped based on laborer/driver/housekeeper, fisherman/farmer, private employees, government employees, students, entrepreneurs, and not working/ housewife /retired. Risk factors are grouped into bisex, hetero, homo, drugs, perinatal, etc. Risk groups are grouped into men sex with men, male sex worker, female sex workers, injecting drug users, shemale, sex worker customers, risky couples, etc.

**Statistical analysis**: The data is presented in frequency and percentage based on a traffic accident history. Chi-square analysis was performed to determine the relationship between the independent and dependent variables. P-value <0.05 was considered statistically significant. The independent variable, which has a p-value lower than 0.25, is included in the multivariable analysis. We analyzed the final model using Binary Regression Logistics. All analyzes were performed by SPSS 22.0 (IBM Corporation, NY, USA).

**Ethical review**: The Health Research Ethics Committee of *Universitas Negeri Semarang* has reviewed and approved the protocol by issuing a letter numbered 038/KEPK/EC/2020. No further ethical clearance is required for the analysis of secondary data.

## Results

Research findings show that the majority of respondents use hospitals as health care facilities for treatment (59.8%), diagnosed with HIV in 2019 (32.2%), domiciled in Semarang (56.4%), aged 15–34 years (54.3%), male (71.6%), not pregnant (95.9%), and working as laborer/driver/housekeeper (10.6%). The majority of respondents have risk factors with homosexual sexual orientation (30.9%) with the MSM group (31.6%) and do not have opportunistic infections (87.7%). The most common opportunistic infections suffered by respondents were tuberculosis (43%), candidiasis (21%), chronic diarrhea (9%), and wasting syndrome (8%) (table 1).

**Table 1:** Characteristics of research respondents

Variable		Number	%
health care facilities	Hospital	814	59,8
	Public health center (puskesmas)	472	34,7
	Community health center (balkesmas)	67	4,9
	Civil Society Organisation (CSO)	9	0,7
year of diagnosis	2019	640	47
	2020	438	32,2
	2021	284	20,9
Region	Semarang	768	56,4
	Outside Semarang	594	43,6
Age	<15 year	20	1,5
	15–34 year	739	54,3
	35–44 year	324	23,8
	45–54 year	209	15,3
	55–64 year	58	4,3
	>64 year	12	0,9
Gender	Male	975	71,6
	Female	387	28,4
pregnancy status	Yes	56	4,1
	No	1306	95,9
Employment	laborer/driver/housekeeper	145	10,6
	fisherman/farmer	10	0,7
	private employees	667	49
	government employees	44	3,2
	Student	74	5,4
	Entrepreneur	32	2,3
	not working/housewife/retired	380	27,9
risk factors	Bisex	30	2,2
	Hetero	872	64
	Homo	421	30,9
	Drugs	11	0,8
	Perinatal	21	1,5
	Etc	7	0,5
risk groups	men seks with men	431	31,6
	male seks worker	10	0,7
	female seks worker	53	3,9
	injecting drug users	10	0,7
	Shemale	9	0,7
	sex worker customer	226	16,6
	risky couple	227	16,7
	Etc	396	29,1
Infection opportunities	Yes	167	12,3
	No	1195	87,7

**Figure 1:**
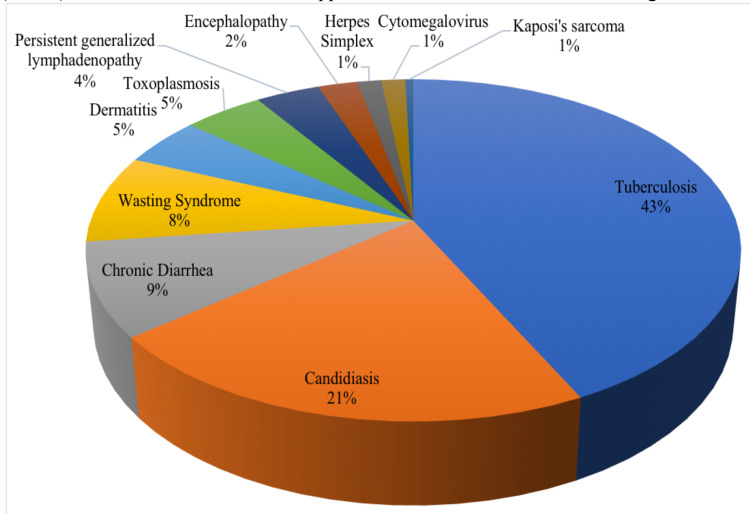
Percentage of Opportunistic Infections in HIV patients

The details distribution of opportunity infections shows in Figure 1.

The results of statistical tests at table 2 showed several variables that were significantly related to opportunistic infections, including health service facilities (p-value 0.001), year of diagnosis of HIV (p-value 0.009), age (p-value 0.001), pregnancy status (p-value 0008 ), occupation (p-value 0.002), HIV risk factors (p-value 0.001), and risk group (p-value 0.001).

**Table 2:** Relationship between risk factor and opportunistic infections in HIV patients

variable		Infection Opportunities				OR (95% CI)	P-value

		No		Yes			

		n	%	n	%		
health care facilities	Hospital	670	56,10	144	86,20	reff	0,001
	Public health center (puskesmas)	453	37,90	19	11,40	0,19 (0,11–0,31)	
	Community health center (balkesmas)	64	5,40	3	1,80	0,21 (0,06–0,70)	
	Civil Society Organisation (CSO)	8	0,70	1	0,60	0,58 (0,07–4,68)	
year of diagnosis	2019	543	45,40	97	58,10	1,77 (1,12–2,80)	0,009
	2020	394	33,00	44	26,30	0,05 (0,03–0,07)	
	2021	258	21,60	26	15,60	reff	
region	Semarang	674	56,40	94	56,30	0,99 (0,71–1,37)	0,978
	Outside Semarang	521	43,60	73	43,70	reff	
age	<15 year	19	1,60	1	0,60	reff	0,001
	15–34 year	682	57,10	57	34,10	1,58 (0,20–12,07)	
	35–44 year	273	22,80	51	30,50	3,55 (0,46–27,10)	
	45–54 year	171	14,30	38	22,80	4,22 (90,55–32,51)	
	55–64 year	40	3,30	18	10,80	8,55 (1,06–68,88)	
	>64 year	10	0,80	2	1,20	3,80 (0,30–47,21)	
gender	male	851	71,20	124	74,30	1,16 (0,80–1,68)	0,469
	female	344	28,80	43	25,70	reff	
pregnancy status	Yes	56	4,70	0	0,00	0,06 (0,01–0,97)	0,008
	No	1139	95,30	167	100,00	reff	
employment	laborer/driver/housekeeper	126	10,60	19	11,70	0,78 (0,45–1,37)	0,002
	fisherman/farmer	7	0,60	3	1,80	2,24 (0,56–8,90)	
	private employees	604	50,80	63	38,70	0,54 (0,37–0,79)	
	government employees	36	3,00	8	4,90	1,16 (0,51–2,62)	
	student	71	6,00	3	1,80	0,22 (0,06–0,72)	
	entrepreneur	26	2,20	6	3,70	1,20 (0,47–3,05)	
	not working/housewife/retired	319	26,80	61	37,40	reff	
risk factors	bisex	731	61,20	141	84,40	reff	0,001
	hetero	27	2,30	3	1,80	0,57 (0,17–1,92)	
	homo	399	33,40	22	13,20	0,28 (0,18–0,45)	
	drugs	11	0,90	0	0,00	0,22 (0,01–3,83)	
	perinatal	20	1,70	1	0,60	0,25 (0,03–1,94)	
	etc	7	0,60	0	0,00	0,34 (0,02–6,06)	
risk groups	men seks with men	407	34,10	24	14,40	reff	0,001
	male seks worker	8	0,70	2	1,20	4,24 (0,85–21,07)	
	female seks worker	50	4,20	3	1,80	1,01 (0,29–3,50)	
	injecting drug users	10	0,80	0	0,00	0,79 (0,04–13,92)	
	shemale	8	0,70	1	0,60	2,12 (0,25–17,64)	
	sex worker customer	178	14,90	48	28,70	4,57 (2,72–7,69)	
	risky couple	196	16,40	31	18,60	2,68 (1,53–4,69)	
	etc	338	28,30	58	34,70	2,91 (1,77–4,78)	

Table 3 show the logistic regression modeling result that show there were 4 models with the last model showing that controlling for other variables, the variables associated with opportunistic infections were year diagnosed (p-value 0.001; AOR 0.678; 95% CI 0.25–0.507), age (p-value 0.001; AOR 1339; 95% CI 1.133–1.582), and risk factors (p-value 0.003; AOR 0.708; 95% CI 0.565–0.886). Age became the most influential variable on opportunistic infection after controlling for other variables.

**Table 3:** The effects of the risk factor on opportunistic infections in HIV patients

Variables	Models				AOR	95% CI	
	
	1	2	3	4		lower	upper
health care facilities	0,000	0,000	0,000	0,000	0,338	0,225	0,507
year of diagnosis	0,003	0,003	0,003	0,001	0,678	0,538	0,854
Age	0,003	0,001	0,001	0,001	1,339	1,133	1,582
Risk factors	0,006	0,006	0,003	0,003	0,708	0,565	0,886
Employment	0,066	0,132	0,200	-	-	-	-
Risk group	0,369	0,254	-	-	-	-	-
pregnancy status	0,997	-	-	-	-	-	-

## Discussion

This study showed 12.3% (n=167) of HIV patients experienced OI, where OI was more common in HIV patients with risk groups for sex workers (28.70%), at-risk couples (18.60%), and MSM individuals (15.40). The most common types of OI were tuberculosis infection (43%), candidiasis (21%), and diarrhea (9%). Previous reports on the prevalence of OI vary (11) reported the prevalence of OI to be 63.9% (n = 954). A study in Vietnam showed the majority of OI among MSM and heterosexual patients were 63.4% and 81.7%, respectively. The most frequent OIs in the MSM group were human papillomavirus (HPV) (11%), followed by hepatitis B virus (8.5%), mycobacterium tuberculosis (7.3%), and talaromycosis (2.4%)(12).

The research findings show that age is the most influential variable on opportunistic infections after controlling for other variables, the age group 45–54 years and 55–64 years being the most at-risk group; this is following research reports in the United States that people with HIV by age group are generally Globally in 2020 increased in people aged 13–24 years, 35–44 years, and 45–54 years. The diagnosis remains stable among people aged 25–35 years and 55 years and over (13,14). This group has risky behavior.

This study found that work is associated with opportunistic infections. A person who does not work or a housewife who has HIV has a significant relationship to the incidence of opportunistic infections. This is following a study in Ethiopia that found that traders and homemakers are one of the risk factors for the incidence of HIV/AIDS (15,16). Another study regarding the incidence of opportunistic infections in People Living With HIV AIDS at Dr. Kariadi Hospital Semarang obtained different results from this study. The study states that work is not a direct cause of opportunistic infections in People Living With HIV AIDS at Kariadi Hospital, Semarang (17).

The other finding in this study shows that another variable that has a relationship with opportunistic infections is heterosexual risk factors. This research is supported by other research conducted in Indonesia. Research conducted in Gresik and Surabaya found that heterosexuality is a significant risk factor for opportunistic infections in HIV patients. (18,19). Research in Vietnam found that the prevalence of opportunistic infections in HIV patients with heterosexuals reached 81.7% (12). Opportunistic infections that can occur in heterosexual patients infected with HIV are fungal infections Cryptococcus neoformans (20).

This study found an association between the MSM group and the clientele of sex workers with increased OI in HIV patients. Unprotected sexual activity through commercial sex is thought to be linked to Sexual Transmitted Infections. The proportion of using condoms was relatively high in the heterosexual group. The prevalence of Sexual Transmitted Infections was low in this subpopulation, suggesting that other infections may be spread through commercial sex engagement. Certain diseases, including HBV, HCV, HPV, and herpes simplex virus, have been shown to be infected through unprotected sex with an infected partner (21). In addition, the prevalence of certain opportunistic infections associated with sexual transmission among sex workers is significantly high (22,23), indicating a high probability of transmission for heterosexual patients using the service. In seeking other studies that investigated this association and demonstrated only the heterosexual group with an abnormal increase in obtaining IO when buying sex, the results may have important implications for optimizing treatment programs for the two subpopulations (12).

This study has several limitations. First, this is a single-center study; the results may not generalize to the different characteristics of people living with HIV, either internationally or in other parts of Indonesia. Second, analysis with secondary data has been carried out at several different times so that there is limited information for consideration in the discussion and that it does not include factors in the therapy program that must be considered as risk factors (ARV use, adherence, etc.) this is due to the unavailability of these data in the SIHA system. The conclusion of this study is that the 45–54 and 55–64 age groups have the most influential relationship with the incidence of OI in HIV patients; therefore, it is necessary to design an appropriate intervention program for this particular subgroup.
